# Identification of an l-Arabitol Transporter from *Aspergillus niger*

**DOI:** 10.3390/biom13020188

**Published:** 2023-01-17

**Authors:** Jiali Meng, Miia R. Mäkelä, Ronald P. de Vries

**Affiliations:** 1 Fungal Physiology, Westerdijk Fungal Biodiversity Institute & Fungal Molecular Physiology, Utrecht University, Uppsalalaan 8, 3584 CT Utrecht, The Netherlands; 2 Department of Microbiology, University of Helsinki, Viikinkaari 9, 00790 Helsinki, Finland

**Keywords:** *Aspergillus niger*, polyol, l-arabitol, transporter, wheat bran, sugar beet pulp

## Abstract

l-arabitol is an intermediate of the pentose catabolic pathway in fungi but can also be used as a carbon source by many fungi, suggesting the presence of transporters for this polyol. In this study, an l-arabitol transporter, LatA, was identified in *Aspergillus niger*. Growth and expression profiles as well as sugar consumption analysis indicated that LatA only imports l-arabitol and is regulated by the arabinanolytic transcriptional activator AraR. Moreover, l-arabitol production from wheat bran was increased in a metabolically engineered *A. niger* mutant by the deletion of *latA*, indicating its potential for improving l-arabitol-producing cell factories. Phylogenetic analysis showed that homologs of LatA are widely conserved in fungi.

## 1. Introduction

Polyols (sugar alcohols) have diverse functions in fungi, such as [1] in storage of the reducing power and coenzyme regulation, [2] to adjust the osmotic pressure by acting as compatible solutes, [3] act as endogenous carbohydrate reserves, [4] act as translocatory compounds and [5] as intermediates of major metabolic pathways [1]. Mannitol is the most common polyol found in fungi, while others include l- and d-arabitol, erythritol, d-threitol, xylitol, galactitol, sorbitol and volemitol [2]. Polyols are widely used in food and pharmaceutical industries with several health-related advantages, such as low-calorie, low-glycemic, low-insulinemic, anticarcinogenic and prebiotic properties [3].

l-arabitol occurs intracellularly as an intermediate in the fungal pentose catabolic pathway (PCP) but is rarely found in high amounts in nature [4]. However, it can be industrially produced from plant biomass derived sugars, and it is therefore widely used in the food and pharmaceutical industries as one of 12 building block chemicals (C3–C6 compounds) derived directly from biomass. Improving l-arabitol production is a major target in industrial biotechnology, focusing, in particular, on fermentation optimization and strain screening and development [5]. 

In the fungal PCP, l-arabinose is reduced to l-arabitol and then converted in two steps to xylitol [6,7]. Studies in *A. niger* showed that xylitol and l-arabitol can both support growth as a sole carbon source similar to d-xylose and l-arabinose [7,8,9,10,11]. Several mutants of *A. niger* constructed by metabolic engineering have been shown to accumulate and secrete xylitol and l-arabitol from d-xylose or l-arabinose [10,12]. These results demonstrate that *A. niger* has the capacity to both take up and secrete these two polyols, indicating the existence of polyol transporters in this fungus.

Reports concerning polyol transporters are limited, but there has been a growing interest in them in recent years. Several polyol transporters have been studied in plants, such as the H^+^/mannitol transporter in celery and the polyol transporter AtPLT5 from *Arabidopsis* [13,14]; in red algae, such as transporters from *Galdieria sulphuraria* [15]; and in bacteria, such as d-arabinitol and ribitol transporters from *Klebsiella pneumonia* [16]. Some characterized fungal transporters are also able to transport polyols, such as hexose transporters Hxt11, Hxt13, Hxt15, Hxt16 and Hxt17 from *Saccharomyces cerevisiae* [17]; two l-arabinose transporters Lat1 and Lat2 from *Ambrosiozyma monospora* [18,19]; and five polyol/H^+^ symporters Sgl1, Stl1, Syi1, Syl1 and Syl2 from *Debaryomyces hansenii* [20]. 

Recently, 86 putative sugar transporter genes were identified in a genome-wide study of the sugar transportome of *A. niger* [21]. These predicted and 61 characterized fungal sugar transporters were phylogenetically classified to nine clades with diverse functional motifs and possible sugar specificity. Clade I contains pentose and glycerol transporters, such as XAT1 [22], Lat2 [18], Xyp29 [23] and Stl1 [24]. Lat2 has transport activities of l-arabinose, l-arabitol and ribitol in *A. monospora* grown on l-arabinose instead of d-glucose [18]. In this study, putative l-arabitol transporter encoding genes from *A. niger* were identified by combining transcriptome and phylogeny analysis. Construction of deletion strains for these genes and subsequent growth profiling revealed that one of them (NRRL3_04757, named *latA*) encodes an l-arabitol transporter. The application of *latA* deletion for l-arabitol production from wheat bran and sugar beet pulp by *A. niger* was also studied. Wheat bran and sugar beet pulp are two feedstock examples that reflect the typical differences between mono- and dicot cell wall composition. While both contain cellulose, wheat bran contains large amounts of arabinoxylan, while sugar beet pulp contains pectin and xyloglucan. These substrates were chosen to evaluate the use of such different feedstocks for l-arabitol production. 

## 2. Materials and Methods

### 2.1. Strains, Media and Growth Conditions

*Escherichia coli* DH5α was used for plasmid construction and was grown on Luria-Bertani (LB) medium supplemented with 50 μg/mL ampicillin (Sigma-Aldrich, Zwijndrecht, The Netherlands). *A. niger* strains used in this study were deposited at the CBS culture collection of Westerdijk Fungal Biodiversity Institute (Utrecht, The Netherlands) with numbers shown in Table 1. The uridine auxotrophic and non-homologous end-joining (NHEJ) deficient *A. niger* strain N593 Δ*ku70* was used as the reference strain. The mutants were generated using CRISPR/Cas9 genome editing [25]. The primers used for creating two deletion mutations are listed in Table S1. *A. niger* protoplasting and transformation were performed as described previously [26]. All *A. niger* strains were grown at 30 °C on Complete Medium (CM) or Minimal Medium (MM) [27] supplemented with required carbon source. For plate cultivations, 1.5% (*w*/*v*) agar (Sigma-Aldrich) was added, and 1.22 g/L uridine (Sigma-Aldrich) was supplemented for auxotrophic strains. A total of 1.3 mg/mL 5-fluoroorotic acid (5-FOA) (Sigma-Aldrich) was added in the solid medium for counter selecting colonies containing the *pyrG* marker gene on ANEp8-Cas9 plasmids. 

*A. niger* strains were grown on CM plates with 1% d-glucose at 30 °C for 5 days. Spores were harvested in ACES buffer (Sigma-Aldrich) and were counted using a haemocytometer (Neubauer, Marienfeld, Germany). Solid MM was used for growth profiles supplemented with 25 mM d-glucose, 25 mM d-xylose (Sigma-Aldrich), 25 mM l-arabinose (Sigma-Aldrich), 25 mM xylitol (Sigma-Aldrich), 25 mM l-arabitol (Sigma-Aldrich), 25 mM d-arabitol (Sigma-Aldrich), 25 mM glycerol (Sigma-Aldrich), 25 mM galactitol (Sigma-Aldrich), 25 mM d-sorbitol (Sigma-Aldrich), 25 mM d-mannitol (Sigma-Aldrich), 25 mM *myo*-Inositol (Sigma-Aldrich), 25 mM adonitol (ribitol) (Sigma-Aldrich) or 25 mM dl-threitol (Sigma-Aldrich). A total of 200 spores in 5 μL ACES buffer were inoculated on the plates and incubated at 30 °C for up to 12 days. 

### 2.2. Expression Analysis

**Figure 1:**
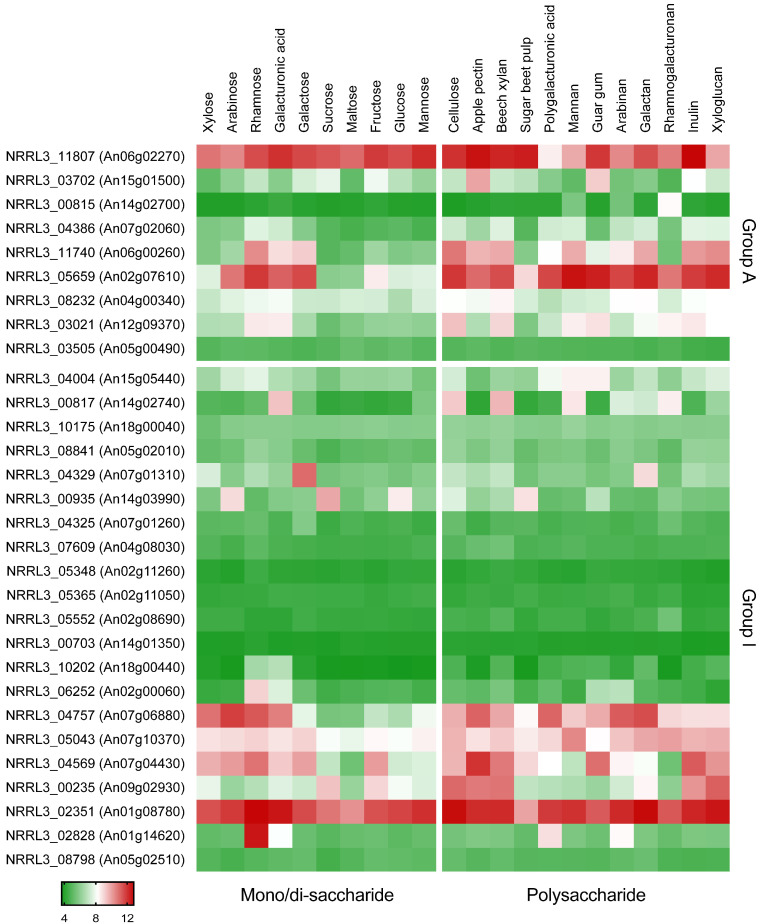
Expression profiles of putative sugar transporters in *A. niger* on diverse carbon sources [21]. Protein IDs of *A. niger* NRRL3 are shown in the figure, and numbers in brackets reflect protein IDs from *A. niger* CBS 513.88. The color code represents averaged and logged expression values (FPKM + 1) of replicates. The heat map was drawn using GraphPad Prism (https://www.graphpad.com/, accessed on 11 January 2023). Group A and I refer to the phylogenetic grouping of transporters, as described previously [21].

Expression analysis was performed on previously obtained micro-array data [21,28]. In brief, the strains were pre-cultured for 16 h in CM with 2% fructose (Sigma-Aldrich), after which the mycelium was harvested, washed with MM without carbon sources and aliquots were transferred to MM with different carbon sources, as indicated in Figure 1. After 2 h, mycelium was harvested and used for RNA isolation, which was then used for micro-array analysis. More details on the procedure can be found in [21,28].

**Table 1:** *A. niger* strains used in this study.

Strain	CBS Number	Genotype	Reference
N593 Δ*ku70*	CBS 138852	*cspA1*, *kusA::amdS*, *pyrG¯*	[29]
Δ*latA*	CBS 147737	*cspA1*, *kusA::amdS*, *pyrG¯*, *latA¯*	This study
Δ05659	CBS 147735	*cspA1*, *kusA::amdS*, *pyrG¯*, 05659¯	This study
Δ*ladA*Δ*xdhA*Δ*sdhA*	CBS 144672	*cspA1*, *kusA::amdS*, *pyrG¯*, *ladA¯*, *xdhA¯*, *sdhA¯*	[10]
Δ*ladA*Δ*xdhA*Δ*sdhA*Δ*latA*	CBS 149003	*cspA1*, *kusA::amdS*, *pyrG¯*, *ladA¯*, *xdhA¯*, *sdhA¯, latA¯*	This study

### 2.3. Transfer and Sugar Consumption Experiments

A total of 1 × 10^6^ spores/mL were inoculated to 250 mL CM with 2% d-fructose in 1 L Erlenmeyer flasks for precultures and incubated in rotary shakers (Infors, Basel, Switzerland) at 30 °C and 250 rpm for 16–18 h. The mycelia were harvested by filtration on Miracloth (Sigma-Aldrich) under sterile conditions and washed with MM. Equal amounts of mycelia were transferred to 50 mL MM in 250 mL Erlenmeyer flasks containing 1% wheat bran or 1% sugar beet pulp and were incubated in rotary shakers at 30 °C, 250 rpm. The transfer experiments were performed in triplicate. In total, 2 mL culture liquid was harvested after 0, 4, 8, 24, 32, 48, 56, 72 and 80 h, and supernatants were stored at −20 °C after centrifugation for measurement of extracellular xylitol and l-arabitol concentrations. The same approach was used for sugar consumption experiments, in which 25 mM l-arabitol, 25 mM l-arabinose, 25 mM xylitol, 25 mM d-xylose, 25 mM galactitol, 25 mM d-galactose and a mixture of 25 mM l-arabitol and 25 mM xylitol were used as the substrates. In total, 2 mL culture liquid was harvested after 0, 1, 2, 4, 8 and 24 h to measure concentrations of monosaccharides and polyols that were used as carbon sources in liquid cultures.

### 2.4. Quantification of Monosaccharides and Polyols 

The culture liquid samples were heated at 95 °C for 15 min and centrifuged for 5 min at 14,000 rpm. The supernatants were 10-fold diluted with MilliQ water (Merck, Amsterdam, The Netherlands) prior to analysis of xylitol and l-arabitol by HPLC (Dionex ICS-5000 + system; Thermo Scientific, Nieuwegein, The Netherlands) equipped with CarboPac PA1 column (2 × 250 mm with 2 × 50 mm guard column; Thermo Scientific), as described previously [30]. All selected monosaccharides and polyols as mentioned above with concentrations of 5–250 μM were used as standards for identification and quantitation. 

### 2.5. Phylogenetic Analysis

Homologs of LatA from other fungi were obtained using BLASTP based on the amino acid sequence of *A. niger* LatA (NRRL3_04757) on the MycoCosm database (https://mycocosm.jgi.doe.gov/mycocosm/home, accessed on 12 February 2022). In this phylogenetic analysis, eight Eurotiomycetes (*A. niger* NRRL3, *Aspergillus nidulans* FGSC A4, *Aspergillus oryzae* RIB40, *Penicillium rubens* Wisconsin 54-1255, *Aspergillus tubingensis* v1.0, *Aspergillus carbonarius* ITEM 5010 v3, *Aspergillus fumigatus* Af293 and *Penicillium subrubescens* FBCC1632/CBS 132785), three Sordariomycetes (*Neurospora crassa* OR74A v2.0, *Trichoderma reesei* QM6a and *Fusarium oxysporum* f. sp. *Lycopersici* 4287 v2), one Leotiomycete (*Botrytis cinerea* v1.0), two Dothideomycetes (*Phaeosphaeria nodorum* SN15 v2.0 and *Pseudocercospora* (*Mycosphaerella*) *fijiensis* v2.0) and two Saccharomycetes (*Candida albicans* SC5314 and *Saccharomyces cerevisiae* S288C) species were selected, and the best hits were used for construction of a phylogenetic tree. The specific l-arabinose transporter Lat2 from *A. monospora* was also included in the phylogenetic analysis [19]. All collected sequences were aligned using MAFFT v7.0 (https://www.ebi.ac.uk/Tools/msa/mafft/) [31]. Phylogenetic analysis was computed using the Neighbor joining method with 500 bootstraps of the Molecular Evolutionary Genetics Analysis (MEGA v7.0, https://www.megasoftware.net/) program [32].

## 3. Results and Discussion

### 3.1. Identification of Putative l-Arabitol Transporters

In a previous study [21], 86 sugar transporters were proposed in *A. niger* CBS 513.88 and 30 predicted transporters grouped to Clade A and Clade I mainly containing inositol/fructose and pentose/glycerol transporters, respectively. The gene NRRL3_04757 (An07g06880) is a homolog of Lat2 of *A. monospora*, which is a characterized l-arabinose transporter that is also able to transport l-arabitol [18,19]. The gene NRRL3_05659 (An02g07610) is the homolog of two polyol/H^+^ symporters Syl1 and Syl2 from *D. hansenii* [20]. We assumed that the uptake of l-arabitol would be co-regulated with l-arabinose catabolism since l-arabitol has been shown to be the inducer of AraR, the l-arabinose-related transcriptional activator [9]. As we already had transcriptome data available for a set of monosaccharides [21], we evaluated this data for the expression profiles of sugar transporter genes on different carbon sources (Figure 1). This showed that NRRL3_04757 and NRRL3_05659 were highly expressed on l-arabinose. While the expression of NRRL_04757 was highest on l-arabinose, NRRL_05659 had the highest expression on l-rhamnose. The expression level of NRRL3_04757 was significantly reduced in single deletion mutants of two transcriptional activators AraR and RhaR, respectively, showing that it is regulated by AraR on l-arabinose and by RhaR on l-rhamnose [21]. Since no other sugar transporters had clear expression patterns pointing to putative l-arabitol transporters, the transporters NRRL3_04757 and NRRL3_05659 were selected as putative l-arabitol transporters.

#### 3.1.1. Deletion of *latA* Resulted in Impaired Growth on l-Arabitol 

**Figure 2:**
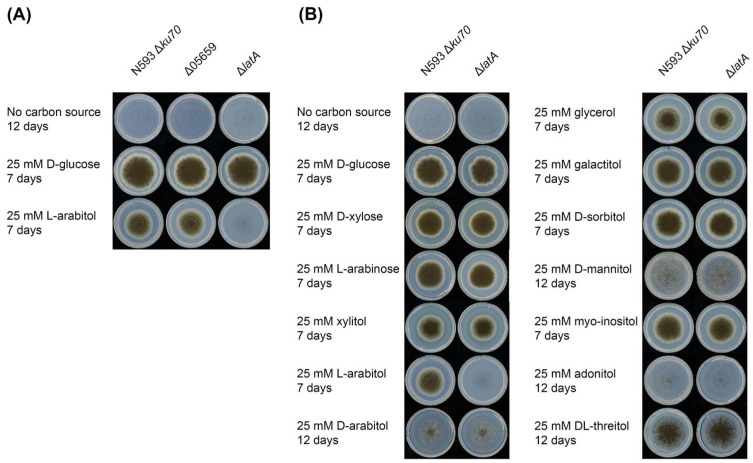
Phenotypic analysis of mutant and reference strains. (**A**) Growth profiling of the *A. niger* reference strain N593 Δ*ku70* and two single deletion mutants Δ05659 and Δ*latA* on l-arabitol. (**B**) Growth profiling of the *A. niger* reference strain N593 Δ*ku70* and Δ*latA* on different sugars and polyols.

To confirm the function of NRRL3_04757 and NRRL3_05659, single deletion mutants were generated in the *A. niger* N593 Δ*ku70* reference strain, and their growth on l-arabitol was compared to the reference strain (Figure 2A). The deletion mutant of NRRL3_04757 (Δ*latA*) showed impaired growth on l-arabitol compared to the reference strain, indicating that NRRL3_04757 is a main l-arabitol transporter under this condition, and the gene was therefore named *latA*. The strong phenotype suggests that there is no redundancy in l-arabitol transport in *A. niger*, which is a clear contrast with the higher number of d-glucose and d-xylose transporters [33,34,35]. A possible explanation for this is that d-glucose and d-xylose are abundantly present in natural carbon sources of *A. niger*, while l-arabitol is an intermediate of the PCP and, therefore, much less abundant in the natural biotope of this fungus. In contrast, the NRRL3_05659 deletion strain grew identical to the reference strain on l-arabitol (Figure 2A), indicating that NRRL3_05659 is not involved in l-arabitol transport.

The effect of LatA on the growth of *A. niger* on several pentoses and polyols was also investigated (Figure 2B). No changes in phenotype were observed on any of the other tested compounds, suggesting that LatA is highly specific for l-arabitol. However, we cannot exclude that transport of (some of) these other polyols could be mediated by multiple transporters, affecting a possible phenotype of Δ*latA* on these substrates. However, this would mean that LatA has a minor contribution to their transport, as at least reduced growth would otherwise be expected. Production of LatA in a heterologous host (e.g., *S. cerevisiae*) could shed more light on the range of compounds it is able to transport. 

In a previous study [21], LatA (NRRL3_04757, An07g06880) was assigned to Sugar Transporter Group I of *A. niger*, containing pentose or glycerol transporters. LatA is the closest homolog of Lat2 of *A. monospora* [21]). Lat2 is capable of transporting l-arabinose [18], but no phenotype on this sugar was observed for the *latA* deletion strain in our study (Figure 2B). As there may be additional l-arabinose transporters in *A. niger*, we cannot exclude at this time that LatA may also contribute to l-arabinose transport. The expression data (Figure 1) showed that *latA* was induced by d-xylose and l-arabinose and regulated by AraR on l-arabinose [21], which could support a role for LatA as a pentose transporter. However, since l-arabitol catabolism is also stimulated by l-arabinose and l-arabitol is an intermediate of this pathway, the expression profile also supports the role as a specific l-arabitol transporter.

#### 3.1.2. Deletion of *latA* Only Affected l-Arabitol Consumption

**Figure 3:**
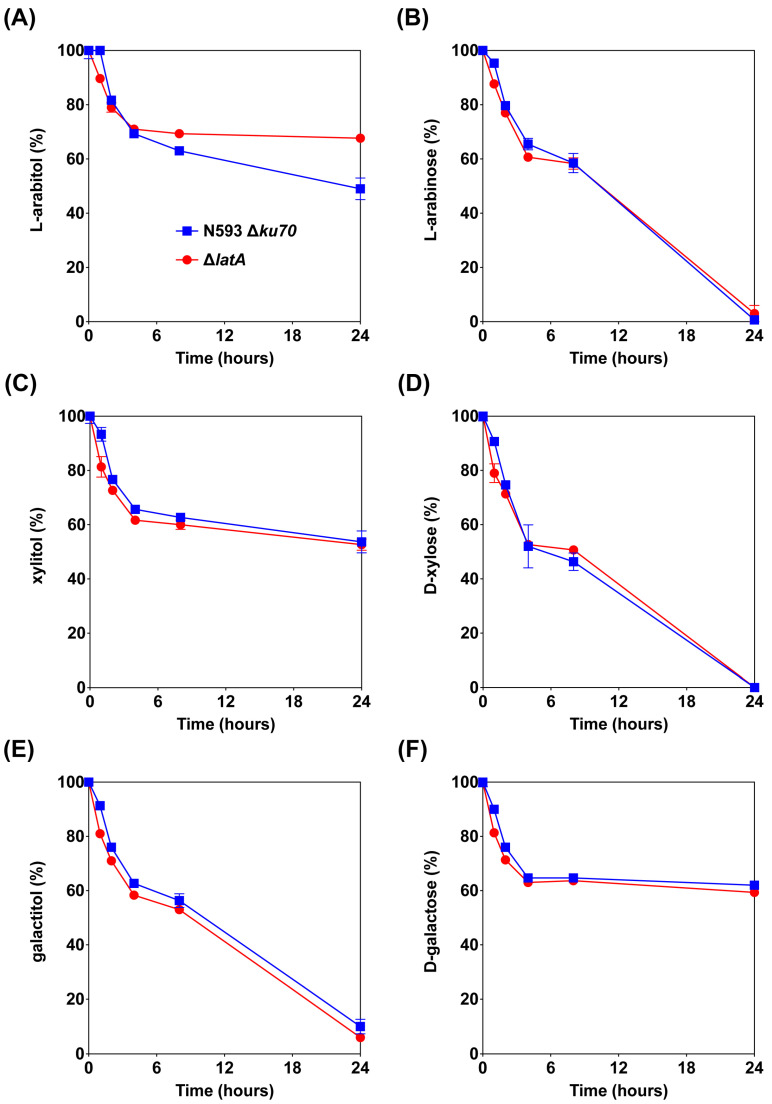
Consumption of sugars and polyols by the *A. niger* reference strain and Δ*latA* in liquid cultures supplemented with l-arabitol (**A**), l-arabinose (**B**), xylitol (**C**), d-xylose (**D**), galactitol (**E**) and d-galactose (**F**) as the substrates. The error bars indicate the standard deviation between biological triplicates.

The sugar consumption of the reference strain N593 Δ*ku70* and the mutant Δ*latA* was also determined in liquid cultures to study the transport function of LatA on l-arabitol, l-arabinose, xylitol, d-xylose, galactitol and d-galactose (Figure 3). An initial decrease in the extracellular concentrations of all compounds was observed in both strains during the first 4 h of cultivation, which is likely due to absorption to the mycelium. At later time points, the deletion of *latA* only had an obvious effect on l-arabitol consumption, as this was almost abolished after 4 h of cultivation in the mutant Δ*latA* compared to the reference strain (Figure 3A). This suggests that LatA is a highly specific l-arabitol transporter and plays a predominant role in l-arabitol utilization in *A. niger* under these conditions. This is also consistent with the findings of growth profiling (Figure 2). The deletion of *latA* did not affect l-arabinose utilization (Figure 3B), so we can exclude a function for LatA in l-arabinose transport.

Moreover, the mixture of l-arabitol and xylitol was also used as the substrate to study whether the presence of both compounds affects each other’s uptake in the reference strain and the mutant Δ*latA* (Figure S1). After the initial reduction in the first 4 h, no l-arabitol was consumed in Δ*latA*, while the presence of xylitol slightly decreased the l-arabitol utilization in the reference strain. The deletion of *latA* did not affect xylitol utilization on the mixture of l-arabitol and xylitol.

#### 3.1.3. The deletion of *latA* Positively Affected l-Arabitol Production from Wheat Bran and Sugar Beet Pulp

**Figure 4:**
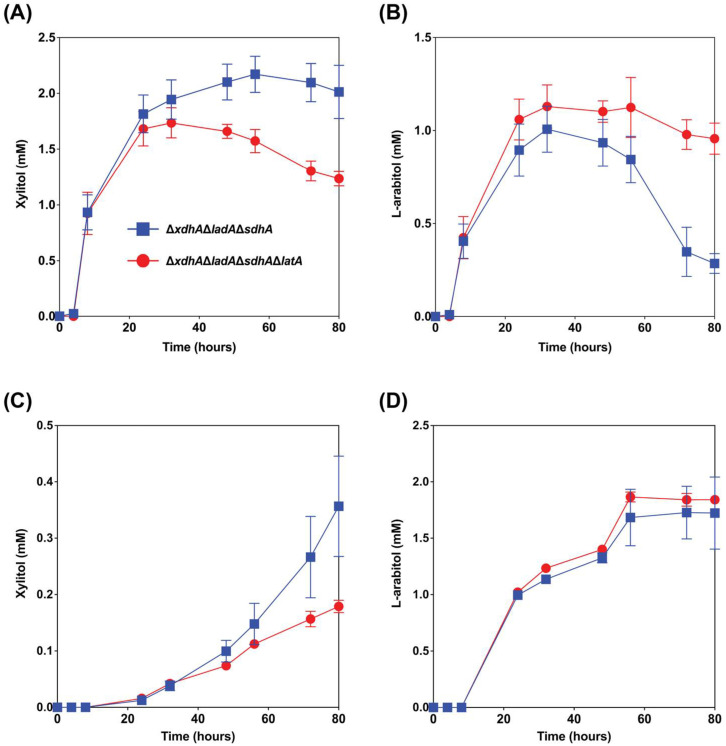
Extracellular xylitol and l-arabitol concentrations from wheat bran ((**A**) and (**B**), respectively) and sugar beet pulp ((**C**) and (**D**), respectively) cultures of the *A. niger* mutant strains Δ*ladA*Δ*xdhA*Δ*sdhA* and Δ*ladA*Δ*xdhA*Δ*sdhA*Δ*latA*. The error bars indicate the standard deviation between biological triplicates.

The reference strain N593 Δ*ku70* barely accumulates l-arabitol during growth on wheat bran, as this is metabolized intracellularly [10,12]. In the Δ*ladA*Δ*xdhA*Δ*sdhA* triple mutant, the conversion of l-arabitol to l-xylulose is blocked, and as a result, this mutant accumulates l-arabitol and xylitol during growth on wheat bran and secretes this [10,12]. However, we observed that after some time, l-arabitol is taken up again by the fungus [12]. Deleting *latA* would potentially prevent re-consumption of l-arabitol by *A. niger* stimulating l-arabitol production in this metabolically engineered strain. To test this, we deleted *latA* in the strain Δ*ladA*Δ*xdhA*Δ*sdhA*. The results showed that the deletion of *latA* in the triple mutant increased the maximum titer of l-arabitol from wheat bran by 12% (Figure 4B). The l-arabitol level decreased in the triple mutant after 32 h of cultivation, most likely by re-consumption, while this was delayed until 56 h when *latA* was also deleted. It confirmed that deletion of *latA* can reduce/delay re-consumption l-l-arabitol when other carbon sources are depleted.

In the previous study, l-arabitol production from sugar beet pulp in the triple mutant was up to 2-fold higher than from wheat bran and other studied crude biomass [12]. The proportion of l-arabinose in sugar beet pulp is higher than in wheat bran, and conversely, the content of d-xylose in wheat bran is significantly higher than in sugar beet pulp (Table 2). This difference in composition of these two substrates can explain why the maximum titer of l-arabitol (2.0 mM) from sugar beet pulp was higher than from wheat bran (1.0 mM) in Δ*ladA*Δ*xdhA*Δ*sdhA* strain. While a small increase in l-arabitol titer from sugar beet pulp was observed when *latA* was also deleted, this is not statistically significant (Figure 4D). The largest effect of the *latA* deletion was in fact seen on wheat bran, where re-consumption of l-arabitol after 60 h was strongly reduced (Figure 4B).

Conversely, the extracellular xylitol titer was decreased by almost 50% after 80 h of cultivation when *latA* was deleted (Figure 4A,C), most likely due to increased re-consumption, possibly because l-arabitol cannot be re-consumed. 

Our results demonstrate that the deletion of *latA* in a xylitol and l-arabitol producing strain has a moderate increase on the l-arabitol concentration, indicating that this gene is a relevant component of a strain engineering approach to generate polyol cell factories.

#### 3.1.4. LatA Is Widely Present in Ascomycete Fungi

**Figure 5:**
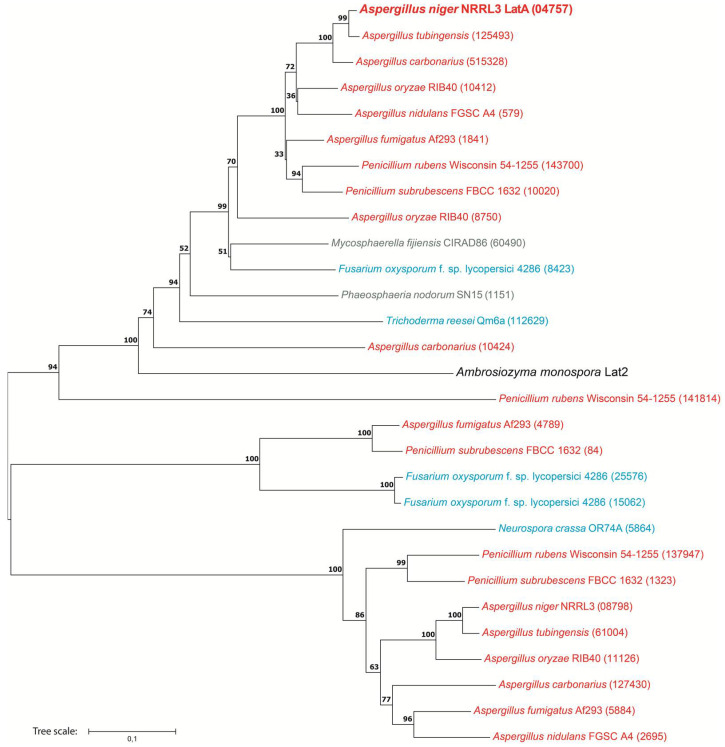
Unrooted phylogenetic tree of homologs of LatA from *A. niger*. The tree includes a specific l-arabinose transporter Lat2 from *A. monospora* (shown in larger font and black), the characterized l-arabitol importer LatA in *A. niger* (shown in larger font and boldface) and homologs of LatA in selected fungi. Bootstrap values are indicated on the nodes, which are supported by the Neighbor joining method. Colors represent the fungal taxonomic groups. Red = Eurotiomycetes, blue = Sordariomycetes, grey = Dothideomycetes. Numbers in brackets reflect the protein ID from JGI MycoCosm [38].

Homologs of LatA are present in most species selected for our analysis, except *B. cinerea*, *C. albicans* and *S. cerevisiae* (Figure 5), indicating that homologs of LatA are widely present across the phylum Ascomycota. The phylogenetic analysis provided many candidate polyol transporters for further characterization in other fungi. Some species contain multiple candidate l-arabitol transporters, perhaps for enhancing the activity to transport polyols [37]. However, care should be taken in assigning function to these homologs, as already, the functional characterization of Lat2 from *A. monospora* [19] suggests differences in substrate specificity between these transporters. 

In conclusion, we identified a highly specific l-arabitol transporter in *A. niger* that contains homologs across Ascomycota and has potential as a target for metabolic engineering of l-arabitol producing fungal cell factories.

## Figures and Tables

**Table 2:** Composition of wheat bran and sugar beet pulp (mol%) (Adapted from [36]).

Feedstock	Rha	Ara	Xyl	Man	Gal	Glc	UA	Total	Polysaccharides
Wheat bran	0.0	16.5	34.6	1.4	1.7	42.5	3.3	53.7	cellulose, arabinoxylan
Sugar beet pulp	1.5	29.0	2.4	2.1	6.5	32.0	27.0	56.0	cellulose, pectin, xyloglucan

Rha = rhamnose, Ara = arabinose, Xyl = xylose, Man = mannose, Gal = galactose, Glc = glucose, UA = uronic acid (glucuronic acid for wheat bran and galacturonic acid for sugar beet pulp).

## Supplementary Materials

The following supporting information can be downloaded at: https://www.mdpi.com/article/10.3390/biom13020188/s1, Table S1: Primers used in this study. Table S2: Composition of wheat bran and sugar beet pulp (mol%). Figure S1: The polyol consumption of the *A. niger* reference strain N593 Δ*ku70* and the mutant Δl*atA* in the liquid culture containing a mixture of l-arabitol and xylitol as the substrate.
