# Supplementary material for: Identification of an l-Arabitol Transporter from Aspergillus niger
Source: Biomolecules. 2023 Jan 17;13(2):188. doi: 10.3390/biom13020188 (PMC9953744; doi:10.3390/biom13020188)

Table S1. Primers used in this study. The guide RNAs (gRNA) for gene deletion are marked in red and the linkers are shown in blue.

Primers	Sequences	Description
P1	CAACCTCCAATCCAATTTGACTCCGCCGAACGT ACTG	For constructing gRNA
P2	ACTACTCTACCACTATTTGAAAAGCAAAAAAG GAAGGTACAAAAAAGC	For constructing gRNA
P3-*latA*	ATGGGCTGTCCGACGCGAAAGACGAGCTTACT CGTTTCG	For constructing gRNA of *latA*
P4-*latA*	TTTCGCGTCGGACAGCCCATGTTTTAGAGCTA GAAATAGCAAG	For constructing gRNA of *latA*
latA 5' Fw	CCTAATCCGCTATCCCAAC	For amplifying *latA* 5' flank and identifying correct deletion mutation
latA 5' Rv	CGATAGCGAATCCTAGCAGTGGTGACTACGCT AGGGTAG	For amplifying *latA* 5' flank
latA 3' Fw	ACTGCTAGGATTCGCTATCGGCACTTTTCTAAT GACTG	For amplifying *latA* 3' flank
latA 3' Rv	CAGATCACCGTACCAGCG	For amplifying *latA* 3' flank and identifying correct deletion mutation
latA 5' NEST Fw	CTGTACCACCCGCTCCAG	For fusion of *latA* 5' and 3' flanks
latA 3' NEST Rv	CTAATTCACAGTCTACATTC	For fusion of *latA* 5' and 3' flanks
P3-NRRL3_05659	CCGTGCAACTCTTGCTTCCTGACGAGCTTACTC GTTTCG	For constructing gRNA of gene NRRL3_05659
P4-NRRL3_05659	AGGAAGCAAGAGTTGCACGGGTTTTAGAGCT AGAAATAGCAAG	For constructing gRNA of gene NRRL3_05659
NRRL3_05659 5' Fw	CTTTCACTTTCAGTCTATCTGG	For amplifying gene NRRL3_05659 5' flank and identifying correct deletion mutation
NRRL3_05659 5' Rv	CGATAGCGAATCCTAGCAGTCCAGGCTTAGGA CGACAG	For amplifying gene NRRL3_05659 5' flank
NRRL3_05659 3' Fw	ACTGCTAGGATTCGCTATCGGTCTATGAGCAC TTCAAG	For amplifying gene NRRL3_05659 3' flank
NRRL3_05659 3' Rv	GGGATTGTAAGAGTGTGC	For amplifying gene NRRL3_05659 3' flank and identifying correct deletion mutation
NRRL3_05659 5' NEST Fw	GGCAAATGCTTACCATCG	For fusion of gene NRRL3_05659 5' and 3' flanks
NRRL3_05659 3' NEST Rv	GGGTCTTTTGTTAGATAC	For fusion of gene NRRL3_05659 5' and 3' flanks

Table S2. Composition of wheat bran and sugar beet pulp (mol%).

	Rha	Ara	Xyl	Man	Gal	Glc	GalUA	Total	polysaccharides
Wheat bran	0.0	**16.5**	**34.6**	1.4	1.7	42.5	3.3	53.7	cellulose, arabinoxylan
Sugar beet pulp	1.5	**29.0**	2.4	2.1	6.5	32.0	27.0	56.0	cellulose, pectin, xyloglucan

Figure S1. The polyol consumption of the *A. niger* reference strain N593 Δ*ku70* and the mutant Δ*latA* in the liquid culture containing a mixture of L-arabitol and xylitol as the substrate. The error bars indicate the standard deviation between biological triplicates.

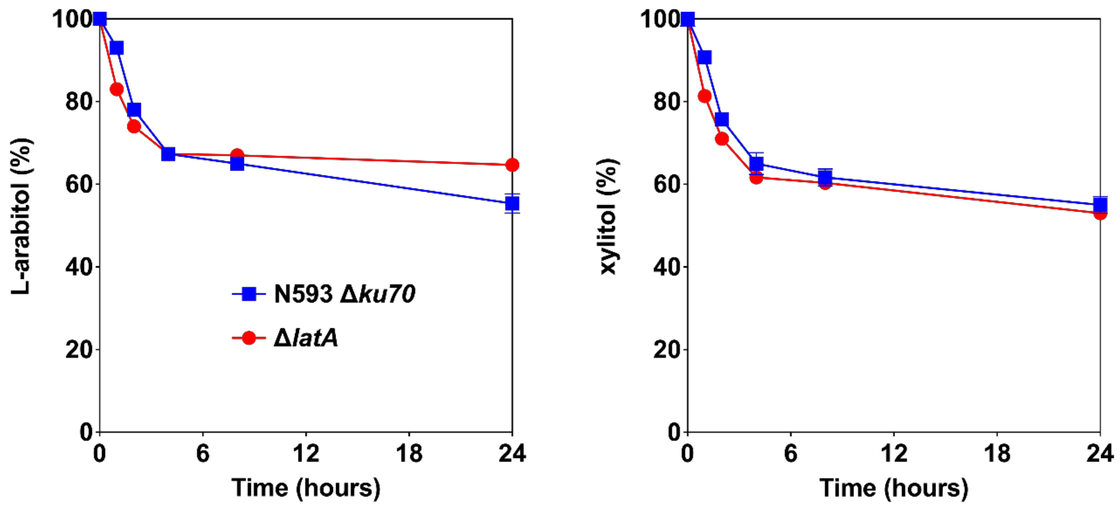

Supplement: Supplementary file 1 [file biomolecules-13-00188-s001.zip › biomolecules-2148955-supplementary.pdf]
